# Comparative assessment of the SjSAP4-incorporated gold immunochromatographic assay for the diagnosis of human schistosomiasis japonica

**DOI:** 10.3389/fpubh.2023.1249637

**Published:** 2023-09-01

**Authors:** Yi Mu, Jonas Rivera, Donald P. McManus, Kosala G. Weerakoon, Allen G. Ross, Remigio M. Olveda, Catherine A. Gordon, Hong You, Malcolm K. Jones, Pengfei Cai

**Affiliations:** ^1^Molecular Parasitology Laboratory, QIMR Berghofer Medical Research Institute, Brisbane, QLD, Australia; ^2^School of Biomedical Sciences, The University of Queensland, Brisbane, QLD, Australia; ^3^Rural Health and Medical Research Institute, Charles Sturt University, Orange, NSW, Australia; ^4^Department of Immunology, Research Institute for Tropical Medicine, Manila, Philippines; ^5^School of Public Health, Faculty of Medicine, The University of Queensland, Brisbane, QLD, Australia; ^6^School of Veterinary Science, The University of Queensland, Brisbane, QLD, Australia

**Keywords:** schistosomiasis, *Schistosoma japonicum*, gold immunochromatographic assay (GICA), POC-CCA, droplet digital PCR, diagnosis, SjSAP4, ASSURED criteria

## Abstract

**Background:**

Schistosomiasis, a disease caused by parasites of the genus *Schistosoma*, remains a global public health threat. This study aimed to validate the diagnostic performance of a recently developed gold immunochromatographic assay (GICA) for the detection of *S. japonicum* infection in a rural endemic area of the Philippines.

**Methods:**

Human clinical samples were collected from 412 subjects living in Laoang and Palapag municipalities, Northern Samar, the Philippines. The presence of *Schistosoma*-specific antibodies in serum samples was tested with the SjSAP4-incorporated GICA strips and the results were converted to fully quantitative data by introducing an *R* value. The performance of the established GICA was further compared with other diagnostic tools, including the Kato-Katz (KK) technique, point-of-care circulating cathodic antigen (POC-CCA), droplet digital (dd) PCR, and enzyme-linked immunosorbent assays (ELISAs).

**Results:**

The developed GICA strip was able to detect KK positive individuals with a sensitivity of 83.3% and absolute specificity. When calibrated with the highly sensitive faecal ddPCR assay, the immunochromatographic assay displayed an accuracy of 60.7%. Globally, the GICA assay showed a high concordance with the SjSAP4-ELISA assay. The schistosomiasis positivity rate determined by the GICA test was similar to those obtained with the SjSAP4-ELISA assay and the ddPCR assay performed on serum samples (SR_ddPCR), and was 2.3 times higher than obtained with the KK method.

**Conclusion:**

The study further confirms that the developed GICA is a valuable diagnostic tool for detecting light *S. japonicum* infections and implies that this point-of-care assay is a viable solution for surveying endemic areas of low-intensity schistosomiasis and identifying high-priority endemic areas for targeted interventions.

## 1. Introduction

Schistosomiasis, a debilitating disease caused by parasites of the genus *Schistosoma*, severely affects the health and socio-economic well-being of more than 250 million people in 78 of the world’s poorest countries (1). In the past three decades, human mass drug administration (MDA), in which the effective oral drug praziquantel was delivered, has been the main strategy for the control of schistosomiasis globally (2). In Asia, hepatosplenic schistosomiasis caused by the zoonotic *Schistosoma japonicum* infection remains prevalent in China, the Philippines, and small foci in Central Indonesia (3). In the Philippines, the transmission of schistosomiasis is year-round due to high year-round precipitation. Added to this, the frequent flooding and strong typhoons, insufficient drug coverage and low compliance with MDA, and the presence of water buffaloes as a major reservoir animal host, meaning that the disease is still highly prevalent in the country. These issues necessitate multi-faceted interventions, such as bovine vaccination, development and implementation of cost-effective diagnostics for rapid mapping and monitoring of the disease in both humans and bovines, along with MDA to provide sustainable control, and beyond, elimination of the disease (4).

Currently, there are a diverse set of diagnostic tools available for the detection of schistosome infections (5–9). Parasitological detection techniques (e.g., the Kato-Katz (KK), urine filtration, and miracidia hatching technique (MHT)), while demonstrating a considerable specificity, had a compromised sensitivity when applied in endemic areas with low prevalence and/or reduced infection intensity of schistosomiasis (5). Improved coprological tests, such as the saline gradient method (10), and Helmintex method (11) showed a higher sensitivity compared with the traditional parasitological procedures; yet usually take longer time in sample processing and are labour-intensive. A variety of immunologic tests such as the circumoval precipitin test (COPT), indirect hemagglutination assay (IHA), the enzyme linked immunosorbent assay (ELISA), and rapid diagnostic tests (RDTs), were cost-effective and widely used during infection control and transmission control stages (8), although may suffer from a relatively low specificity if the crude extracted antigens were incorporated and it is difficult to distinguish between past and current infections. In addition, molecular detection methods mainly based on using polymerase chain reaction (PCR) and its derivative techniques, including nested PCR, real-time quantitative (q)PCR (12–15), droplet digital (dd) PCR assays (16–18), loop-mediated isothermal amplification (LAMP) (19–21), recombinase polymerase amplification (RPA) (22–24), and recently clustered regularly interspaced short palindromic repeats (CRISPR)-assisted diagnostic tests (25, 26) are promising tools for the detection of schistosomes; however at present, the prospect of large scale application of these methods in field areas remains obscure due to their relatively high cost.

The new roadmap for NTDs 2021–2030 recently released by the WHO (27) and revised guidelines for schistosomiasis (28) emphasised the need to develop and evaluate diagnostic tools to facilitate surveillance, control and elimination strategies (29). The WHO is seeking expert consultation on diagnostics with a particular focus on Point-of-care (POC) tests (30). Current antigen detection (AgD)-based POC tests for schistosomiasis are based on the probing of proteoglycan components present in the gut vomit of juvenile and adult worms known as circulating anodic antigens (CAAs) or circulating cathodic antigens (CCAs) using lateral flow assays (31–33). The POC-CCA assay is a commercially available cassette test that has been widely used for the detection of active *S. mansoni* infection in both Africa and South America (11, 34–36). However, the assay showed less potential for the diagnosis of other *Schistosoma* species (37–39). In addition, the assay suffers some pitfalls, such as cross-reactivity, underperformed specificity, and a “trace” reading issue (34, 38). The up-converting phosphor-lateral flow CAA (UCP-LF CAA) assay can detect all *Schistosoma* species quantitatively, exhibiting more accurate diagnostic performance than the POC-CCA assay. While the UCP-LF CAA assay has been suggested to be a promising tool for the diagnosis of low-intensity schistosome infections (40), it requires a large volume of samples, an additional filter device and concentration step and a special UCP-LF strip reader (41), and is, as of writing, not available as a commercial test currently.

To date, a number of antibody detection (AbD)-based lateral flow immunochromatographic assay (LFIA) strips have been developed for the rapid diagnosis of schistosomiasis japonica (42–45). However, these crude antigen-incorporated assays were found to be cross-reactive with other helminths. Recently, we developed a novel gold immunochromatographic assay (GICA) strip incorporating a recombinant saposin protein of *S. japonicum*, rSjSAP4, an unprecedented antigen for serological diagnosis of schistosomiasis (46–48). A preliminary assessment of the GICA cassette showed its potential in the rapid screening of schistosomiasis japonica (49). In this study, we further assess and validate the performance of the developed GICA cassettes with a large sample size, and calibrate its performance with the KK technique, ddPCR, POC-CCA and in-house ELISA assays that have been employed for the detection of *S. japonicum* infection against the same human cohort as reference (18, 46).

## 2. Materials and methods

### 2.1. Ethics approval and consent to participate

The human research ethics approval for conducting this study was obtained from the Institutional Review Board of the Research Institute for Tropical Medicine (RITM), Manila, the Philippines (number 2015–12) and the Human Research Ethics Committee, QIMR Berghofer Medical Research Institute (QIMRB), Brisbane, Australia (Ethics Approval: P524). Written informed consent was received from each study participant (written informed consent was obtained from their legal guardians for those aged 15 years and under).

### 2.2. Sample collection, processing, and storage

Human clinical samples (faeces, serum, urine, and saliva) were collected from 412 subjects from 18 barangays in Palapag and Laoang, Northern Samar, the Philippines (18, 46, 47). Individual stool samples (10–15 g) were collected from each participant. Two faecal samples were sought from each individual on different days within a week for the KK analysis. The remainder of the first faecal sample (~10 g) was fixed in 80% ethanol. Blood sample (~10 mL) was collected from each individual with serum separation tubes. The blood samples were allowed to clot for 30 min at ambient temperature, and serum samples were then collected after centrifugation at 1500 × *g* for 10 min. Spot urine samples (~30 mL) were collected into 50 mL Falcon tubes and stored at 4°C. Saliva (~2 mL) was collected into a 5 mL centrifuge tube using the passive drool method under the supervision of a well-trained medical technologist. All processed samples were stored at 4°C and transported on wet ice to the RITM, Manila, where the samples were stored at −20°C. Subsequently, all samples were shipped to QIMRB, Brisbane, Australia, on dry ice. Faecal samples were then stored at 4°C, while serum, urine and saliva samples were stored at −80°C, for further analysis. Serum samples collected from healthy donors (*n* = 23) residing in a non-endemic area for schistosomiasis japonica (Qiqihar, Heilongjiang Province, China), were used as controls.

### 2.3. Parasitological detection

The KK slides were examined by experienced technicians at the RITM. For each stool sample, three KK thick smear slides were prepared and examined under a light microscope. Infection intensity for *S. japonicum* was defined as the number of eggs per gram of faeces (EPG). In order to improve the accuracy of the KK analysis, 10% of slides were randomly selected and subjected to re-examination by an experienced microscopist.

### 2.4. Preparation and measurement of the GICA strips

The GICA strips were developed by Zoonbio Biotechnology (Nanjing, China) (49). Briefly, colloidal gold particles with a mean particle diameter of 70 nm were used to coat recombinant protein rSjSAP4. The gold-rSjSAP4 conjugate was suspended in the buffer containing 20 mM Tris, 5% (w/v) sucrose, and 2.5% (w/v) trehalose at a final concentration of 10 μg/mL, and dispensed onto conjugate pad (glass fibre membrane) at a volume of 35 μL/cm. The pad was dried in a biochemical incubator for 12 h at 37°C. By using an XYZ Biostrip Dispenser (HM3030, Shanghai Kinbio Tech. Co., Ltd., Shanghai, China), protein G (1 mg/mL) (Zoonbio Biotechnology, Nanjing, China) and mouse anti-His tag mAb (0.7 mg/mL) (Zoonbio Biotechnology, Nanjing, China) were dispensed onto the nitrocellulose (NC) membrane (CN140, Sartorius, Goettingen, Germany) at a volume of 1 μL/cm to form the test and control lines, respectively. The NC membrane was then dried at room temperature in a biochemical incubator for 6 h. The absorbent pad (filter paper), coated NC membrane, conjugate pad, and sample pad, were laminated and pasted onto a plastic-backed support card with a 1–2 mm overlap. The assembled scale board was cut lengthwise into strips measuring 3 × 60 mm using a guillotine cutter (ZQ2002, Shanghai Kinbio Tech. Co., Ltd., Shanghai, China). The resulted strips were placed in a plastic cassette, which was further packaged into a silica gel desiccant-containing aluminium foil bag, and stored at room temperature.

The GICA strips were initially tested on 40 KK-positive [KK (+)] subjects, 20 KK-negative [KK (−)] and 20 control individuals in a previous pilot study (49). For the current study, the GICA cassettes were further tested for comparison with the other diagnostics following our previous pilot study protocol (49). Briefly, for each test, 50 μL diluted serum sample (1:20) was added to each cassette which was scanned at 10 min after sample loading. All images were further analysed by a Java-based image processing program, ImageJ to determine an *R* value, which was defined as the intensity of the test (*T*) line divided by that of the corresponding control (*C*) line, converting the results into fully quantitative data. The tests were determined as invalid when the control band did not appear or when the tests were left to develop for more than 15 min. GICA cassettes from the same batch were used for testing all the serum samples.

### 2.5. Comparative analysis using the KK, ddPCR, POC-CCA, and ELISA assays as references

The performance of the GICA was further calibrated using other diagnostic tests, including the KK, POC-CCA (37), two ELISA assays (Sj23-LHD-ELISA and SjSAP4-ELISA) (46), and four ddPCR assays performed on feces, serum, urine, and saliva, which were designated as F_ddPCR, SR_ddPCR, U_ddPCR, and SL_ddPCR, respectively (18), as references.

### 2.6. Statistical analysis

All statistical analyses were performed using GraphPad Prism version 9 software (GraphPad Software, Inc., California, United States). For analysis of differences in the *R* values between the control group and groups with variable *S. japonicum* egg burdens, one-way ANOVA followed by Holm-Sidak multiple comparison or the Mann–Whitney *U* test was used. A cut-off *R* value was set for the GICA assay with the maximization of Youden index (*J*) on testing of 108 KK (+) individuals and 23 healthy controls. McNemar’s test^1^ was used to determine the differences between the sensitivities determined by the GICA and the other diagnostic methods on testing 108 KK (+) individuals, and the differences between the positivity rates obtained with the GICA and other diagnostics across the different groups stratified by egg burdens. Calibrated by the different reference tests, sensitivity, specificity, positive predictive value (PPV), negative predictive value (NPV) and accuracy were analyzed for the developed GICA. Agreement between the GICA and the other diagnostics assays was determined using the Kappa statistic,^2^ The strength of agreement was measured according to the κ value scores divided into: <0, no agreement; 0.00–0.20 slight agreement; 0.21–0.40 fair agreement; 0.41–0.60 moderate agreement; 0.61–0.80 substantial agreement; and 0.81–1.00 perfect agreement (50). Pearson’s correlation coefficient (*r*) was used to assess the correlation between the immunochromatographic assay and the SjSAP4-ELISA assay for the KK (+) individuals and the entire cohort.

## 3. Results

### 3.1. Study population

The target population comprised 412 subjects [male: *n* = 218 (52.9%), 39.2 ± 16.1 years; female, *n* = 194 (47.1%), 41.5 ± 15.1 years] from a rural schistosomiasis-endemic area, Northern Samar, the Philippines. The human cohort had a moderate schistosomiasis japonica prevalence (26.2%) but a low intensity of infection based on the KK analysis on faecal samples (6 slides on two stool samples), according to the categorization by WHO. Detailed information of the study cohort can be found in previous studies (18, 51, 52).

### 3.2. Transforming the GICA into a fully quantitative assay by introducing an *R* value

**Figure 1:**
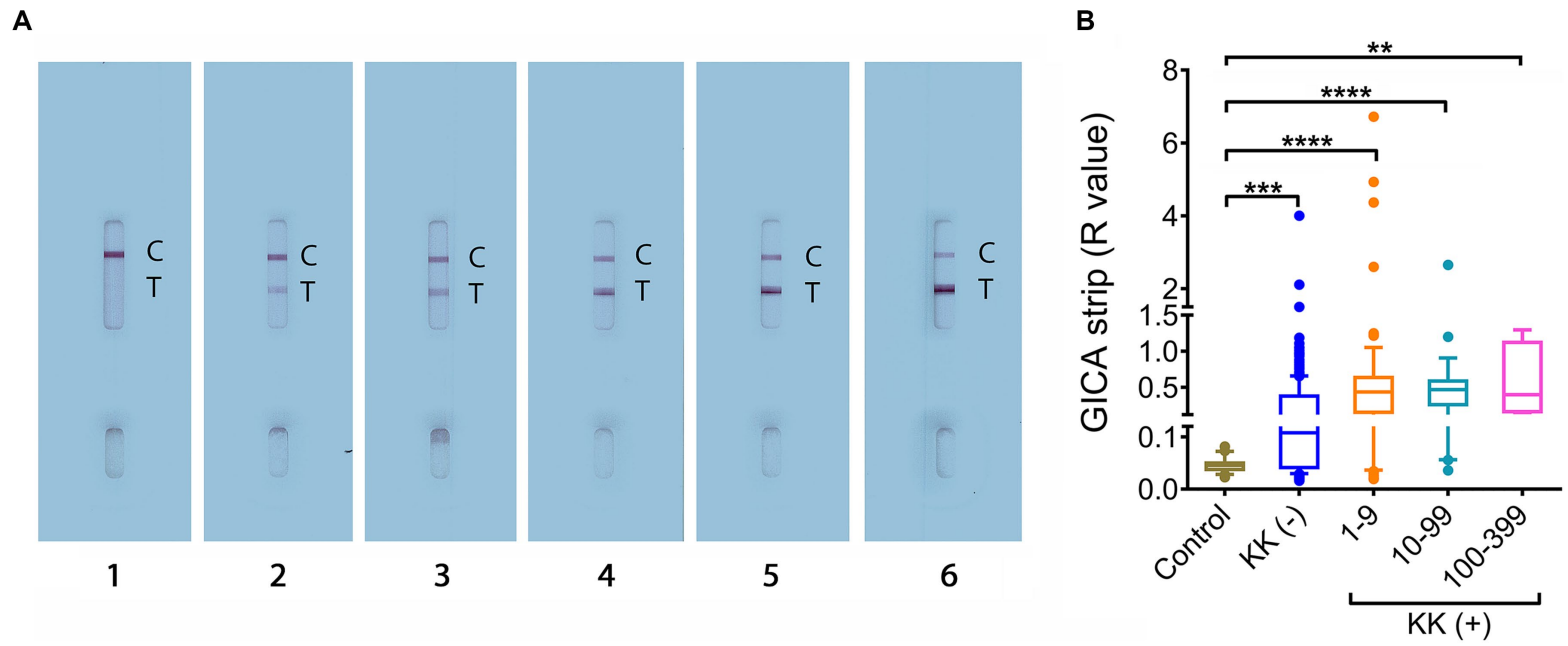
*R* value analysis for the developed GICA cassettes. **(A)** GICA strips showing different ranks of *R* values. Lanes 1–6, strips displaying an *R* value between 0–0.1, 0.1–0.5, 0.5–1, 1–2, 2–3, and > 3, respectively; **(B)** The distribution of *R* values in the controls, KK (−) individuals and different KK (+) groups. (Controls, *n* = 23; KK (−), *n* = 304; KK (+), *n* = 108; EPG 1–9, *n* = 78; EPG 10–99, *n* = 26; EPG 100–399, *n* = 4). Boxes represent the interquartile range of the data with lines across the boxes indicating the median values. The hash marks below and above the boxes indicate the 10th and 90th percentiles, respectively. *p* values were calculated using Kruskal-Wallis test (ns = no significant difference, **, *p* < 0.01;***, *p* < 0.001, ****, *p* < 0.0001).

The GICA cassettes were tested using optimized conditions (i.e., PBS was used as the dilution buffer and serum samples were diluted at a dilution of 1:20) (49). For each GICA cassette, the result was converted into an *R* value, which was calculated by dividing the band intensity of the “T” line with that of the corresponding “C” line. Figure 1A shows GICA cassettes displaying different levels of *R* values. The difference in *R* values between the controls and the target cohort stratified by different infection intensities were further assessed. The *R* values were significantly higher in groups with 1–9 EPG (*n* = 78, *p* < 0.0001), 10–99 EPG (*n* = 26, *p* < 0.0001) and 100–399 EPG (*n* = 4, *p* < 0.01), as well as the KK (−) individuals (*n* = 304, *p* < 0.001) compared with the non-endemic controls (*n* = 23) (Figure 1B).

### 3.3. Diagnostic performance of the GICA cassettes in KK (+) individuals

**Figure 2:**
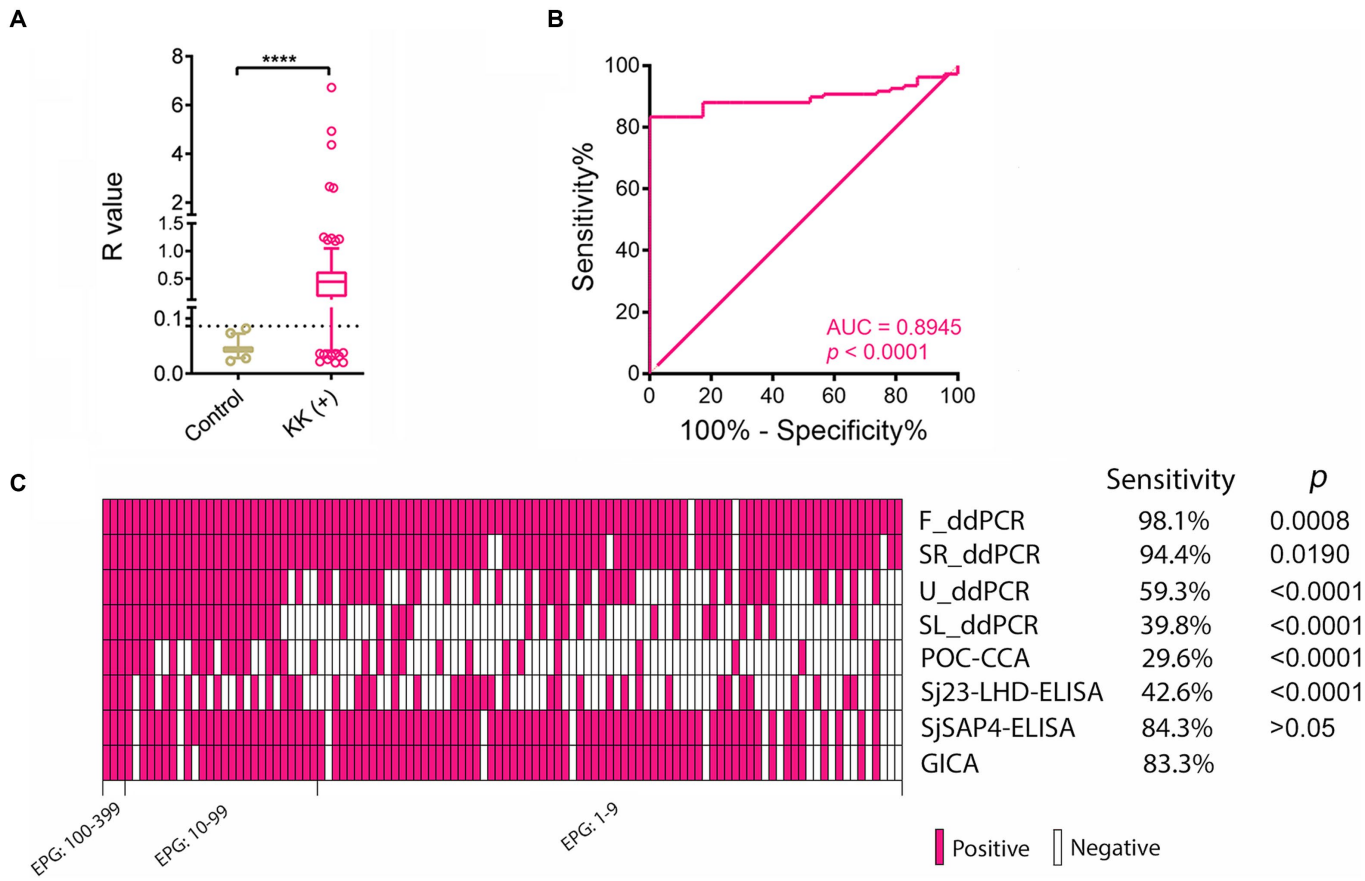
The performance of the SjSAP4-incorporated GICA cassettes in diagnosing the KK (+) individuals. **(A)** Scatter plots showing the *R* values of the non-endemic controls (*n* = 23) and KK (+) subjects (*n* = 108). Boxes represent the interquartile range of the data with lines across the boxes indicating the median values. The hash marks below and above the boxes indicate the 10th and 90th percentiles, respectively. Dashed line: *R* cut-off value. *p* value was determined using the Mann–Whitney *U* test (****, *p* < 0.0001); **(B)** Receiver operating characteristic curve (ROC) analysis was performed to assess the capability of the GICA assay in discriminating the non-endemic controls (*n* = 23) and KK (+) subjects (*n* = 108); **(C)** Clustered results for the eight diagnostics on testing the KK (+) individuals (*n* = 108). Samples are sorted from left to right in a decreased order of egg burden. The differences in sensitivity were compared between the GICA and other diagnostic methods. *p* values were determined by McNemar’s test.

We further analysed diagnostic performance of the GICA strips in the detection of KK (+) subjects (*n* = 108). The *R* values in KK (+) group were significantly higher than those of the healthy controls (*p* < 0.0001) (Figure 2A). When an *R* cut-off value was set at 0.0864, which maximised the Youden’s *J*-index, the GICA strip showed a sensitivity of 83.3% and absolute specificity. The ROC analysis revealed that the established GICA had an AUC level of 0.8945 (*p* = 0.0001) (Figure 2B). The sensitivity of the GICA was significantly lower than these of the F_ddPCR and SR_ddPCR tests (98.1%, *p* = 0.0008 and 94.4%, *p* = 0.019, respectively), but higher than these of the U_ddPCR, SL_ddPCR, POC-CAA and Sj23-LHD-ELISA assays (59.3%, 38.9%, 29.6%, and 42.6%, respectively, *p* < 0.0001 in all comparisons) (Figure 2C).

### 3.4. Positivity rate analysis

We then compared the positivity rate determined by the GICA with those obtained with other diagnostic tests in the detection of *S. japonicum* infection (Table 1). In the subgroup with moderate infection (EPG: 100–399), all the diagnostics had absolute positivity rate. In the subgroup with EPGs between 10–99, the positivity rate assessed by the GICA (88.5%) was significantly higher than those provided by the Sj23-LHD-ELISA (53.8%, *p* = 0.0077) and the POC-CCA cassette (57.7%, *p* = 0.0433), yet no difference was observed when it was compared with those determined by the other diagnostics. In subjects with an extremely low infection intensity (EPG: 1–9), the positivity rate determined by the GICA strips (80.8%) was significantly lower than that obtained with the F_ddPCR test (97.4%, *p <* 0.0001), but significantly higher than those provided by the U_ddPCR, Sj23-LHD-ELISA, SL_ddPCR and POC-CCA assays (47.4%, 35.9%, 24.4%, and 16.7%, respectively, *p* < 0.0001 in all comparisons). In the KK (−) subjects, the positivity rate determined by the GICA (53.8%) was only significantly lower than that judged by the F_ddPCR test (66.1%, *p* < 0.0001) (Table 1). The clustered results for the eight molecular and immunological diagnostics on testing the KK (−) individuals (*n* = 304) are shown in Supplementary Figure S1. The global positivity rate of the target cohort provided by the GICA (60.9%) was significantly lower than that obtained with the F_ddPCR test (74.5%, *p* < 0.0001), but higher than those deduced from the U_ddPCR (47.6%, *p* = 0.0001), SL_ddPCR (25.5%, *p <* 0.0001), Sj23-LHD-ELISA (24.5%, *p* < 0.0001), and POC-CCA (12.4%, *p* < 0.0001) assays. There were no differences in positivity rates obtained with the GICA test and the SjSAP4-ELISA assay in the different subgroups and the entire cohort, respectively. Similarly, no differences were found between the positivity rates determined by the immunochromatographic cassettes and the SR_ddPCR test in all different groups.

**Table 1:** Positivity rates of schistosomiasis japonica determined by different diagnostics in the different subgroups and entire cohort, respectively.

Diagnostic test	KK (+) moderate	KK (+) light^§^				KK (−)	Entire cohort		
	(EPG: 100–399)	(EPG: 10–99)		(EPG: 1–9)		(EPG: 0)		(EPG: 0–399)	
	Positive, % (*n*/*n*)	Positive, % (*n*/*n*)	p*	Positive, % (*n*/*n*)	p*	Positive, % (*n*/*n*)	p*	Positive, % (*n*/*n*)	p*
GICA^†^	100 (4/4)	88.5 (23/26)		80.8 (63/78)		53.0 (161/304)		60.9 (251/412)	
F_ddPCR	100 (4/4)	100 (26/26)	>0.05	97.4 (76/78)	0.0036	66.1 (201/304)	0.0011	74.5 (307/412)	<0.0001
SR_ddPCR	100 (4/4)	100 (26/26)	>0.05	92.3 (72/78)	>0.05	57.6 (175/304)	>0.05	67.2 (277/412)	>0.05
U_ddPCR	100 (4/4)	88.5 (23/26)	>0.05	47.4 (37/78)	<0.0001	43.4 (132/304)	0.0273	47.6 (196/412)	0.0001
SL_ddPCR	100 (4/4)	76.9 (20/26)	>0.05	24.4 (19/78)	<0.0001	20.4 (62/304)	<0.0001	25.5 (105/412)	<0.0001
POC-CCA^†^	100 (4/4)	57.7 (15/26)	0.0433	16.7 (13/78)	<0.0001	6.3 (19/304)	<0.0001	12.4 (51/412)	<0.0001
Sj23-LHD-ELISA^#^	100 (4/4)	53.8 (14/26)	0.0077	35.9 (28/78)	<0.0001	18.1 (55/304)	<0.0001	24.5 (101/412)	<0.0001
SjSAP4-ELISA^#^	100 (4/4)	92.3 (24/26)	>0.05	80.8 (63/78)	>0.05	56.3 (171/304)	>0.05	63.6 (262/412)	>0.05

^§^Individuals with a light infection were arbitrarily divided into two subgroups with EPG of 10–99 and 1–9, respectively. ^†^ *R* cut-off value for the developed GICA: 0.0864; *R* cut-off value for the POC-CAA assay: 0.1344 (37). ^#^OD_450nm_ cut-off values for ELISA assays: Sj23-LHD-ELISA, 0.2185; SjSAP4-ELISA, 0.1832 (46). *p values were determined by McNemar’s test.

**Figure 3:**
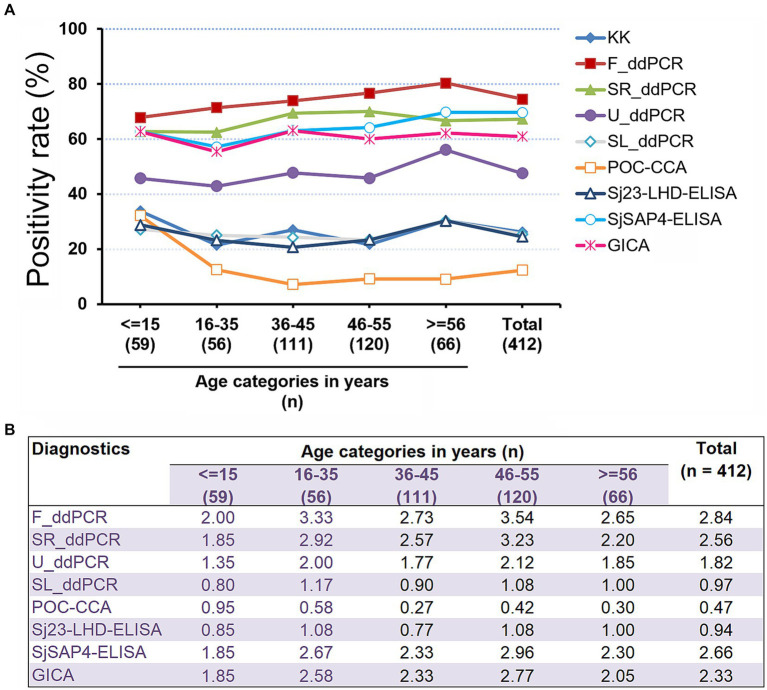
Schistosomiasis japonica positivity rates in the different age groups. **(A)** The schistosomiasis positivity rates determined by all nine diagnostic tests for the human cohort stratified by different age groups. *R* cut-off value for the developed GICA: 0.0864; *R* cut-off value for the POC-CAA assay: 0.1344 (37); OD cut-off values for the ELISA assays: 0.2185 (Sj23-LHD-ELISA) and 0.1832 (SjSAP4-ELISA) (46). **(B)** Fold changes in schistosomiasis positivity rates obtained with the eight molecular and immunological diagnostics vs. that determined by the KK for the investigated cohort stratified by different age groups.

We further analysed the schistosomiasis positivity rates in the different age groups determined by the nine diagnostics (Figure 3). Positivity rates deduced from five diagnostics, the F_ddPCR, SR_ddPCR, U_ddPCR, SjSAP4-ELISA, and GICA strips were higher than that determined by the KK in all age groups (Figure 3A). The positivity rate determined by the developed GICA for each age group was between 1.85 and 2.77 times higher than that assessed by the KK procedure (Figure 3B). The overall schistosomiasis positivity rate of the cohort determined by the established GICA was comparable with those assessed by the SjSAP4-ELISA and SR_ddPCR, and was about 2.3 times higher than that obtained with the KK technique (Figure 3).

### 3.5. Performance of the developed GICA strip with the other diagnostics as references

By employing various diagnostic tools as reference, we further evaluated the diagnostic performance, including sensitivity, specificity, PPV, NPV, and accuracy for the developed GICA strips (Table 2). The GICA test showed the highest sensitivity (92.0%) when the SjSAP4-ELISA assay detecting the serum IgG against the same antigen, SjSAP4, was used as the reference. When the different ddPCR tests were employed as reference tests, the established GICA showed a similar sensitivity between 62.8–66.7%. The developed immunochromatographic assay showed the highest accuracy (92.5%) when the SjSAP4-ELISA assay was adopted as the reference. When calibrated with the F_ddPCR test, the SjSAP4-GICA showed an accuracy of 60.7%. The immunochromatographic GICA displayed a perfect agreement with the SjSAP4-ELISA assay (κ = 0.840), a fair agreement with the KK technique (κ = 0.213) and Sj23-LHD-ELISA assay (κ = 0.214), and a slight agreement with the other diagnostics (κ < 0.2) (Table 2).

**Table 2:** Performance of the GICA using different diagnostic tests as reference.

GICA^†^	Reference test		% Sensitivity (95% CI)	% Specificity (95% CI)	% PPV (95% CI)	% NPV (95% CI)	% Accuracy (95% CI)	Kappa index (95% CI)
	**+**	**−**						
	KK							
**+**	90	161	83.3 (74.9–89.8)	47.0 (41.3–52.8)	35.9 (29.9–42.1)	88.8 (82.9–93.2)	56.6 (51.6–61.4)	0.213 (0.143–0.283)
**−**	18	143						
	F_ddPCR							
**+**	198	53	64.5 (58.9–69.9)	49.5 (39.6–59.5)	78.9 (73.3–83.8)	32.3 (25.2–40.1)	60.7 (55.8–65.4)	0.119 (0.026–0.213)
**−**	109	52						
	SR_ddPCR							
**+**	177	74	64.0 (57.8–69.6)	45.2 (36.6–54.0)	70.5 (64.5–76.1)	37.9 (30.4–45.9)	57.8 (52.8–62.6)	0.087 (−0.010–0.183)
**−**	100	61						
	U_ddPCR							
**+**	123	128	62.8 (55.6–69.5)	40.7 (34.1–47.6)	49.0 (42.7–55.4)	54.7 (46.6–62.5)	51.2 (46.3–56.1)	0.035 (−0.058–0.128)
**−**	73	88						
	SL_ddPCR							
**+**	70	181	66.7 (56.8–75.6)	41.0 (35.5–46.8)	27.9 (22.4–33.9)	78.3 (71.1–84.4)	47.6 (42.7–52.5)	0.053 (−0.02–0.126)
**−**	35	126						
	POC-CCA^†^							
**+**	39	212	76.5 (62.5–97.2)	41.3 (36.2–46.6)	15.5 (11.3–20.6)	92.6 (87.3–96.1)	43.2 (38.4–48.1)	0.066 (0.016–0.116)
**−**	12	149						
	Sj23-LHD-ELISA^#^							
**+**	86	165	85.2 (76.7–91.4)	47.0 (41.3–52.7)	34.3 (28.4–40.5)	90.7 (85.1–94.7)	56.3 (51.4–61.2)	0.214 (0.147–0.281)
**−**	15	146						
	SjSAP4-ELISA^#^							
**+**	241	10	92.0 (88.0–95.0)	93.3 (88.1–96.8)	96.0 (92.8–98.1)	87.0 (80.8–91.7)	92.5 (89.5–94.8)	0.840 (0.786–0.894)
**−**	21	140						

^†^*R* cut-off value for the GICA strip: 0.0864; *R* cut-off value for the POC-CCA assay: 0.1344 (37). ^#^OD_450nm_ cut-off values for the serum ELISA assays: Sj23-LHD-ELISA, 0.2185; SjSAP4-ELISA, 0.1832 (46).

### 3.6. Correlation analysis

**Figure 4:**
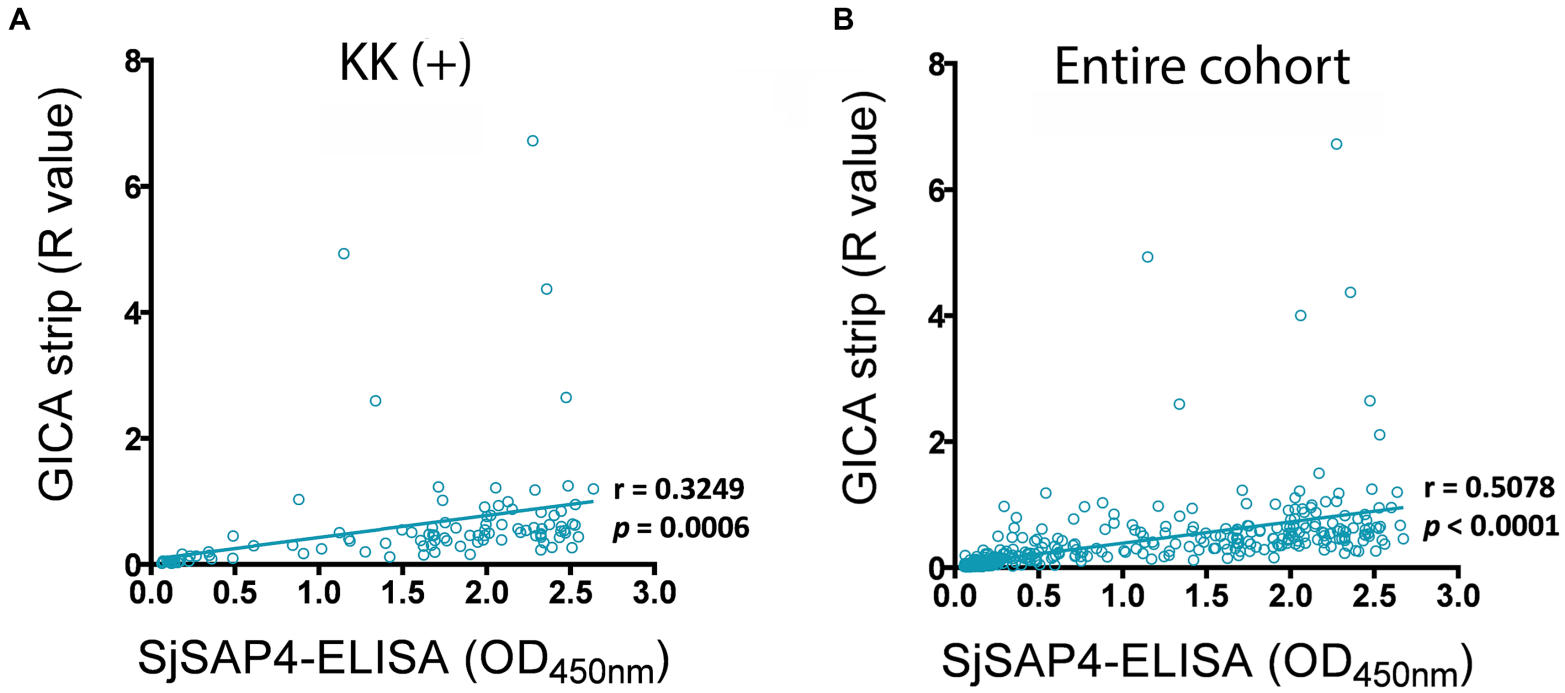
Correlation analysis for the GICA and ELISA assays. Correlations between the GICA test and the SjSAP4-ELISA assay on testing the **(A)** KK (+) individuals (*n* = 108) and **(B)** whole cohort (*n* = 412) using Pearson’s correlation coefficient.

The associations between the developed GICA test and SjSAP4-ELISA assay were investigated in the KK (+) subjects (*n* = 108) and the entire study cohort (*n* = 412). Within the KK (+) subgroup, there was a significant positive correlation (*r* = 0.3249, *p* = 0.0006) between the GICA test and the SjSAP4-ELISA assay (Figure 4A). When analysing the entire cohort, a higher significant correlation (*r* = 0.5078, *p* < 0.0001) was observed between the two assays (Figure 4B).

## 4. Discussion

Schistosomiasis continues to be a major public health problem in the developing world. It is encouraging that it has been successfully controlled in a few of countries, such as China, which is steadily moving towards the goal of schistosomiasis japonica elimination (53). However, in the Philippines, a focal survey performed in 2017 showed that there were still 435 barangays having a high prevalence of human schistosomiasis of >5% (54). Currently, the Philippines government administers MDA in schistosomiasis endemic areas annually through the Schistosomiasis Control and Elimination Program (55). The currently used parasitological approaches, primarily the KK, are not sensitive enough for the diagnosis of schistosomiasis in endemic areas with reduced intensities due to MDA implementation (5). Thus, it is appropriate that the WHO’s 2021–2030 roadmap to eliminate NTDs includes a focus on the development of improved diagnostics for schistosomiasis (27). In this study, we assessed the performance of the recently developed SjSAP4-incorporated GICA test by probing samples collected from a well-defined Philippines cohort, using eight other diagnostics as references.

As stated previously, the sensitivity of the KK technique is compromised in areas with lower infection intensities (51). In the KK (−) individuals, high positivity rates were obtained with both molecular methods, the F_ddPCR (66.1%) and SR_ddPCR (57.6%), and immunodiagnostic tests, the GICA (53%) and SjSAP4-ELISA (56.3%), indicating the KK failed to detect many light infections. This corresponded with a total prevalence, determined by the above mentioned molecular and immunodiagnostic assays, that was 2.33 to 2.84 times higher than that obtained with the KK (26.2%) (Figure 4B). Nevertheless, the KK is still useful for determining infection intensities and prevalence in moderate to high endemic areas, when combined with multiple slides across different days, can be used as a useful standard for comparing other diagnostics. In this study, when tested on the serum samples from KK (+) subjects (*n* = 108), the SjSAP4-incorporated GICA elicited 83.3% sensitivity and absolute specificity, with an AUC value of 0.8945 (Figure 2), indicating a “very good” diagnostic accuracy (56). Although the sensitivity has been decreased from 95% in testing a small subset of these KK (+) subjects (*n* = 40) in a previous study (49), to 83.3% in the current study as the sample size increases, these results indicate that the newly developed GICA strip has strong diagnostic potential in identifying light *S. japonicum* infections, with its performance competitive with the recently developed point of care immunochromatographic test (POC-ICT) incorporated with MS3_01370 antigen for the diagnosis of urogenital schistosomiasis (44).

Molecular methods, such as PCR assays, have been widely used in the detection of schistosome infections and showed high diagnostic performance (5). Although the substantial costs of required reagents and equipment and the requirement of well-trained personnel limit their application in screening of schistosomiasis in resource-poor endemic areas (5), PCR assays can be used as reference tests, which could be more accurate than the traditional parasitological detection procedures, for the evaluation of other laboratory developed diagnostics. For instance, the F_ddPCR and SR_ddPCR assays showed a higher sensitivity compared with those of the POC-CCA, GICA and ELISA assays in identifying the KK (+) individuals (Figure 2C). The developed GICA had a sensitivity of 62.8–66.7% when calibrated with ddPCR tests (Table 2). This relatively low sensitivity could arise as no accurate cut-off values have been set for the ddPCR assays, which may incur some false positives. Furthermore, the relatively lower sensitivity of serological tests compared with PCR, can be also due to some individuals having an abnormally reduced humoral immune response, as seen in previous studies (57). The concordances between the GICA and ddPCR tests are also very poor (κ = 0.053–0.119) (Table 2). This is probably due to the differences in test targets, i.e., ddPCR analysis detects the parasite-derived DNA in samples while the GICA probes host antibodies against SjSAP4. Nevertheless, it is impressive that the SjSAP4-incorporated GICA strip showed 60.7% accuracy when the F_ddPCR assay was employed as a reference test (Table 2). PCR tests, unlike AbD based assays, can be used to determine infection intensities. In our previous study, we found that the ddPCR results, presented as the target gene copy number index (CNI) showed a high degree of correlation with KK-determined EPGs (18), indicating ddPCR assays can measure the CNI as a direct quantitative measure of parasite burden. In contrast, either in field observation by an operator or converting to an *R* value, the AbD based GICA tests cannot reliably differentiate infection intensities. Falsely attributing past infections as active infections occurs for a much longer period of time for serological based techniques compared with molecular methods performed on faecal samples (58). This may contribute to the fact that the SjSAP4-GICA has a relatively low specificity when calibrated with the F_ddPCR test as standard.

Unsurprisingly, the SjSAP4-GICA recorded similar outcomes to the SjSAP4-ELISA. Overall, the developed GICA assay exhibited a diagnostic performance commensurate with that of the SjSAP4-ELISA. There was no significant difference in the positivity rate for the GICA assay and the SjSAP4-ELISA in the group of individuals with an EPG of 10–99 (88.5% vs. 92.3%, *p* > 0.05), an EPG of 1–9 (80.8% vs. 80.8%, *p* > 0.05) and in the KK (−) individuals (53.0% vs. 56.3%, *p* > 0.05) (Table 1); similarly, the prevalence of schistosomiasis japonica determined by the GICA assay and SjSAP4-ELISA assay was comparable (60.9% vs. 65.5%, *p* > 0.05), which was about 2.5-fold higher than that determined by the KK method (26.2%) (Figure 4B). Also, the SjSAP4-incorporated GICA strip recorded the highest sensitivity (92.0%) and accuracy (92.5%) and near-perfect agreement (κ = 0.840) when the SjSAP4-ELISA was used as the reference test (Table 2). When using the F_ddPCR as reference, the GICA assay and the SjSAP4-ELISA assay test displayed similar performance in sensitivity (64.5% vs. 67.4%), specificity (55.2% vs. 47.6%), accuracy (60.7% vs. 62.4%) and agreement (0.119 vs. 0.132) (Table 2; Supplementary Table S1). In this study, we converted the GICA test into a fully quantitative test, which enabled us to assess the correlation between the GICA and SjSAP4-ELISA. The two assays showed significant positive correlations in the KK (+) individuals and the entire human cohort. All these observations indicate that the two assays had a high concordance, which is logical as both are AbD based assay targeting the same antigen. Nevertheless, compared with the rapid GICA, the classic ELISA assay is tedious and labor-intensive, has stringent equipment requirements, and needs well-trained personal to perform.

The POC-CCA assay has been extensively evaluated for the diagnosis of schistosomiasis mansoni in different endemic areas (59–62). In *S. mansoni* infections with a prevalence level of less than 50% (based on the KK procedure), the POC-CCA assay exhibits a 1.5–6 fold higher prevalence than that determined by the KK (63). Of the diagnostics available, it has been suggested that the POC-CCA test best fulfils the ASSURED (A = affordable by the affected individuals; S = sensitive; S = specific; U = user-friendly; *R* = rapid turn-around time and robust performance (e.g., reagents tolerate tropical climate); E = equipment-free; and D = delivered to those in need) criteria for in resource-limited settings (41). However, the diagnostic performance of the assay is *Schistosoma* species-dependent. For instance, in the detection of *S. mekongi* in endemic areas of Lao PDR, the POC-CCA showed a low sensitivity (24.1%) (64). Also, the POC-CCA test had a moderate sensitivity of 63.3%, in the diagnosis of *S. japonicum* patients with infection intensities of EPGs ≥10 (37), while showed a poor sensitivity (29.6%) in identifying *S. japonicum* infection in individuals with extremely low egg burdens (EPG: 1–9) (37). Furthermore, the prevalence determined by the POC-CCA was the lowest among all diagnostics investigated here (Table 1), and only half of that determined by the KK and one in fifth of that deduced by the GICA (Figure 4B). The poor diagnostic sensitivity of the POC-CCA for low intensity *S. japonicum* infections stems from a number of reasons, which has been discussed in a previous study, such as species septicity of capturing antibody incorporated in the POC-CCA cassette (37). Additionally, a relatively high *R* value has been set to retain a considerable specificity due to the concerned issues of low specificity (34) and cross-reactivity with other helminths (65), resulting in a reduced sensitivity for the assay. In all, it can be concluded that the POC-CCA is unsuitable for the diagnosis of schistosomiasis japonica in its current format. In contrast, the relatively high performance of the established GICA would, therefore, be an upgrade for schistosomiasis japonica diagnosis.

In this study, all the tested GICA cassettes were scanned and analysed *in silico*, i.e., a Java-based image processing program, ImageJ, was used to convert results into quantitative *R* values. This would reduce variability from an operator’s training or visual acuity and, also, limit system errors from colour development times and sample absorbance rates (49). Essentially, the ImageJ *R* values would allow fair comparisons between multiple studies with different cassette batches/types. In a rapid field screening, the images of tested GICA strips can be captured by smartphones on-site, and uploaded to a computer for real-time analysis using ImageJ software. However, in some applications where a conversion of an *R* value is inaccessible, the diagnostic performance exhibited here may not be well reflected in those scenarios. Accordingly, other readout methods, such as a semi-quantitative visual scoring method using a color interpretive card as a reference, need to be developed for the interpretation of the GICA. Alternatively, a chromogenic rapid test reader can also be employed to quantify the intensities of bands appearing on the “T” line of the GICA cassettes, providing a quantitative readout. In this regard, parallel studies that assess the agreement and/or correlation between the results determined by ImageJ and those obtained with other readout methods, would, therefore, be of substantial value. Interestingly, Schary et al. recently develop an open-source, all-in-one smartphone-based system for quantitative analysis of lateral flow assays (LFAs) (66). The system includes an *R* Shiny software package with similar function to the Image J for image editing, analysis, data extraction, calibration and quantification of the assays (66). Further combining the smartphone-based *R* Shiny software will increase the potential of the GICA test in the field application.

The current study has the following limitations: (1) The Standards for Reporting Diagnostic accuracy studies (STARD) rules recommend using a “reference test” to evaluate the accuracy of a newly developed diagnostic test (67). However, none of the reference assays employed in this study can be a gold standard test. The KK technique has previously been referred to as a gold standard for intestinal schistosomiasis testing; but the KK lacks sensitivity in low-intensity infections. Further, the Sj23-LHD-ELISA and POC-CCA displayed insufficient sensitivity in the diagnosis of schistosomiasis japonica. And the Sj23-LHD-ELISA and SjSAP4-ELISA have an inherent limitation in the discrimination of current versus previous infections. As regards ddPCR assay, studies have demonstrated the higher sensitivity of this technique compared to conventional qPCR assay (68, 69). The ddPCR assay developed for schistosomiasis diagnosis was able to detect as little as 0.05 fg of template genomic DNA, much less than that in conventional PCR using the same primers, i.e., 0.1 pg (16). The SR_ddPCR, U_ddPCR, and SL_ddPCR assays detect the parasite-derived cfDNA in body fluids, while the F_ddPCR targets schistosome DNA derived from viable and/or decayed eggs and possibly the cell-free DNA released from worms and eggs in faecal samples. The ultra-sensitive nature of ddPCR assay may cause the problem of false positives due to cross-contamination of the samples, assay specificity and/or the detection of a past infection (70). In addition, no stringent CNI cut-off values have been set for these ddPCR assays, a fact that may magnify the problem (18). (2) As a new diagnostic approach, the developed GICA was only tested with serum samples collected from one endemic region. Further evaluations of the assay in different *S. japonicum*-endemic areas with variable intensities of infection and with a much larger sample size will be important in verifying the generalization. (3) The SjSAP4-incorporatd GICA cassettes were tested with only one serum dilution, which means that an optimal serum dilution remains to be determined for the assay.

## 5. Conclusion

The newly developed GICA was able to identify KK (+) subjects in a Philippine cohort with 83.3% sensitivity and 100% specificity. Targeting the whole cohort, a comparison of the GICA with other diagnostic methods further revealed the performance of this immunochromatographic test. In terms of diagnostic performance, the SjSAP4-GICA is comparable to the SjSAP4-ELISA assay but superior to U_ddPCR, SL_ddPCR, POC-CCA and Sj23-LHD-ELISA assays. While F_ddPCR and SR_ddPCR tests may perform better than the GICA strips, these PCR tests are time-consuming and labor-intensive, and requires expensive reagents and equipment. Currently, the SjSAP4-GICA is between $1.5–4.0 per test, depending on the order volume. Overall, the developed GICA stands to meet the ASSURED criteria the best, but further optimization steps are still required. A potential application scenario of the SjSAP4-GICA is its use as the first approach of the two-step diagnostic process suggested by the WHO for schistosomiasis, i.e., started with a high sensitivity test for screening and followed with a second, high specificity test for confirmation (71). Integration of the developed GICA cassettes into the current surveillance strategies in *S. japonicum* endemic areas may help identify high-priority areas for targeted interventions.

## Data availability statement

The original contributions presented in the study are included in the article/Supplementary material, further inquiries can be directed to the corresponding author.

## Ethics statement

The studies involving humans were approved by the Institutional Review Board of the Research Institute for Tropical Medicine (RITM), Manila, the Philippines and the Human Research Ethics Committee, QIMR Berghofer Medical Research Institute (QIMRB), Brisbane, Australia. The studies were conducted in accordance with the local legislation and institutional requirements. Written informed consent for participation in this study was provided by the participants’ legal guardians/next of kin.

## Author contributions

YM, DM, and PC conceptualized the study design and directed the project. YM, JR, KW, RO, and PC developed the methodology. KW, DM, RO, AR, and PC contributed to the acquisition of clinical sample resources. YM, JR, KW, CG, HY, MJ, and PC analyzed, reviewed and interpreted the data. YM, JR, and PC drafted the original manuscript. KW, AR, CG, HY, MJ, and PC revised the manuscript. DM and PC contributed to the funding acquisition. PC supervised the project. All authors contributed to the article and approved the submitted version.

## Funding

This study was supported by the National Health and Medical Research Council (NHMRC) of Australia (IDs: APP1160046, APP2008433, APP1102926, APP1037304, and APP1098244). DM was a NHMRC Leadership Fellow and Senior Scientist at QIMRB. The funders had no role in study design, data collection and analysis, decision to publish, or preparation of the manuscript.

## Conflict of interest

The authors declare that the research was conducted in the absence of any commercial or financial relationships that could be construed as a potential conflict of interest.

## Publisher’s note

All claims expressed in this article are solely those of the authors and do not necessarily represent those of their affiliated organizations, or those of the publisher, the editors and the reviewers. Any product that may be evaluated in this article, or claim that may be made by its manufacturer, is not guaranteed or endorsed by the publisher.

## Footnotes

^1^https://www.graphpad.com/quickcalcs/McNemar1.cfm

^2^https://www.graphpad.com/quickcalcs/kappa1/
