# Supplementary figures and images for: Comparative assessment of the SjSAP4-incorporated gold immunochromatographic assay for the diagnosis of human schistosomiasis japonica
Source: Front Public Health. 2023 Sep 1;11:1249637. doi: 10.3389/fpubh.2023.1249637 (PMC10509475; doi:10.3389/fpubh.2023.1249637)

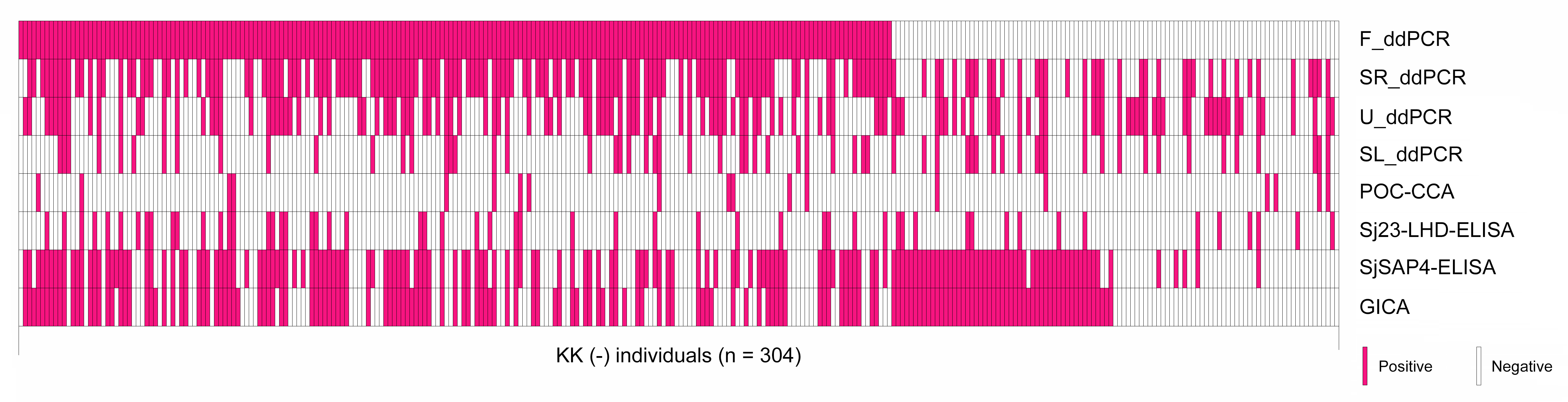

Supplement: Supplementary file 2 [file Image_1.TIF]
